# Knowledge, attitudes, beliefs, behaviour and breast cancer screening practices in Ghana, West Africa

**Published:** 2012-02-17

**Authors:** Samuel Yaw Opoku, Martin Benwell, Joel Yarney

**Affiliations:** 1 Department of Radiography, School of Allied Health Sciences, College of Health Sciences, University of Ghana; 2 Department of Radiography, School of Health, London South Bank University; 3 National Radiotherapy Center, Korle Bu Teaching Hospital, Accra. Ghana

**Keywords:** Breast cancer, screening, mammography, Knowledge, attitude, belief, Ghana, Africa

## Abstract

**Background:**

Late presentation has been observed as the hallmark of breast cancer in Ghanaian women where over 60% of patients report with either stage 3 or 4 of the disease. This cross-sectional study aimed at exploring breast cancer related knowledge and practices in order to develop an appropriate socio-economic and cultural specific model to improve breast cancer care in Ghana.

**Methods:**

The study which was conducted in Accra and Sunyani in Ghana used both quantitative and qualitative methods and employed the theory of planned behavior as a communication and educational model. Information was collected from 474 women using questionnaires. In addition semi-structured interviews were conducted on 10 breast cancer patients; 10 breast clinic attendants; 3 Oncology Consultants and 2 herbalists.

**Results:**

Generally, the respondents displayed knowledge deficit about the disease. However, higher levels of education was associated with better appreciation of the disease (rs =0.316, N465, p < 0.001). The respondents’ attitudes include fear of the disease which was linked to death in most cases; denial and guilt; as well as supernatural attributes. The self-reported breast cancer screening rate (BSE 32%, CBE 12% and mammogram 2%) was poor, however, higher educational of the respondents was very significant for breast cancer screening practices.

**Conclusion:**

The study found that routine mammography screening is not feasible in Ghana at the moment which therefore requires a different approach

## Background

Generally, cancer remains a low priority for 75% of the world population from the developing world that will have to grapple with infectious diseases, the HIV/AIDS epidemic, poverty and malnutrition [1,2]. However, according to the International Association of Research on Cancer [3] of the World Health Organization, recent global cancer statistics indicate a rising global incidence of breast cancer and the increase is occurring at a faster rate in populations of the developing countries that previously enjoyed a low incidence of the disease [4–6] In addition to the fact that the incidence of the disease appears to be on the increase, late presentation with poor outcomes of treatment is the hallmark of breast cancer in most developing countries including Ghana. The 5-year survival of breast cancer in Ghana is less than 25%, compared with over 70% in Western Europe and North America [7–10]. It also disturbing that the average age at diagnosis for breast cancer in Ghana is 46.29 years with a range of 26 to 80 years as compared to an average age of over 65 years in Europe and America [8,10].

Health interventions and programmes are more successful when they are grounded in the appropriate health behaviour change models [11]; therefore this study settled on the theory of planned behaviour as a communication and educational model to help improve breast cancer early detection and treatment and thus improve the overall disease outcome in Ghana. The model centres on factors which lead to a specific intention to act, or behavioural intention, which the theory situates between the attitudes and behaviour [12–14].

### Purpose of the Study

The study determined population-based rates of reported breast cancer screening and assessed breast cancer-related knowledge, attitudes, beliefs among Ghanaian women and to explore their relation to screening practices in the study areas. From the study, appropriate strategies would then be developed with the ultimate aim of reducing the high mortality from breast cancer in Ghana.

## Methods

A cross-sectional descriptive study was carried out in two Ghanaian cities, Accra and Sunyani. The study which was conducted in two phases in 2007 employed both quantitative and qualitative methods of data collection [15].

With a 95% confidence level and a sampling error of 3% and a sample power of 80%, a sample size of 500 was obtained using a standard formula for estimating sample size (Altman, 1991- Nomogram for calculating power size). A cluster, or area, random sampling was used in addition to systematic sampling. Accra was divided into 8 clusters (along geographic boundaries) and cluster was assigned a digit and the 4 clusters were randomly picked from the 8 clusters. For each of the 4 clusters, a sample frame was developed from a database that had been produced by the Maternal and Child Health Services Division (MCH). Using probability, the names and contact details of 350 women aged between 40 and 70 years were selected from the sample frames in the 4 areas in Accra as potential respondents. This modified type of cluster sampling was adopted so that every unit in the population would have the same chance of being selected. The same process was also used to select 150 women from Sunyani. A total sample size of 500 was used.

The quantitative data was collected from 474 Ghanaian women with a questionnaire which was adapted from the Toronto Breast Self Examination Inventory and Champion's breast self examination questionnaire [16]

Significance testing of differences was conducted using the chi-squared test for nominal data and Mann Whitney U test for ordinal data. The quantitative data established very important themes which were further explored by interviews that enhanced a better understanding of the quantitative results. A one-to-one semi structured interviews were conducted on 10 breast cancer patients; 10 breast clinic attendants; 3 medical Consultants involved in breast cancer care in Ghana and 2 traditional healers who claimed to have a cure for breast cancer. The interview data was analysed by constant comparison method which was designed to generate grounded theories from the transcriptions [17,18]. The protocol for the study was approved by the Ethical and Protocol Review Committee of the University of Ghana Medical School.

## Results

### Respondents' main sources of health information and choice of health care

**Figure 1:**
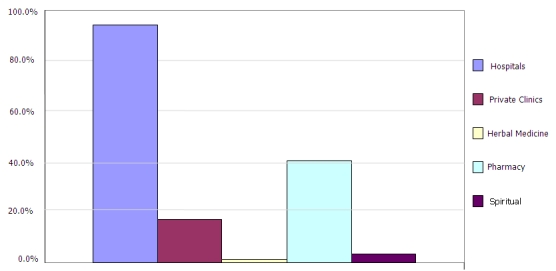
Respondents choices of medical/Health care

The respondents' main sources of health information in general and breast cancer in particular, strongly suggested exposure to multiples sources, but in all respects, the mass media (radio, television, and newspapers) was the major source, totalling 65.4% as shown in Table 1. Because of the supernatural explanation given to cancer in some cases, traditional healers, herbalists and spiritualists have become an important health care outlet used in Ghana as shown in Figure 1.

**Table 1:** Participants main sources of health information

N=474	Number that use the source	Percentage that use the source
Radio	187	39.8
Television	97	20.5
Nurses/Midwives	66	13.9
Doctors	59	12.4
Churches	37	7.8
Friends	31	6.5
Newspapers/Magazines	24	5.1
Women's Groups	23	4.7
Family Members	15	3.2

### Incidence of Breast Cancer in Ghana

On perception about the incidence of the disease in Ghana, 35.7% of the respondents described the disease as very common, 24.5% thought it was common and 12.8% described it as uncommon whilst 27% did not know.

### Level of Education and their Self-reported Knowledge of Breast Cancer

The respondents displayed a knowledge deficit about both breast cancer and breast cancer screening which was evident from the poor appreciation of the risk factors and high level of misconceptions and misinformation. For example only 8.2% and 1.7% mentioned increasing age and early menarche respectively as risk factors. Others factors mentioned included smoking (6.3%), obesity (1%), late menarche (1.5%), family history (5%) and benign breast disease (4.2%). As a result, the disease is still associated with myths and uncertainty surrounding its causes and risk factors. A prominent misconception held by 20% of the respondents was to the effect that coins put in the brassieres can increase a woman's risk for the disease. However, in all these, a weak correlation was found between the levels of education and the self-reported knowledge about the disease, with higher levels of education being associated with higher levels of appreciation about the disease (r_s_ =0.316, N465, p <0.001) as shown in Table 2.

**Table 2:** Factors mentioned by respondents as breast cancer risks

Response	Number	Percentage
Increasing Age	37	8.2
Female sex	19	4. 0
Family History	39	8 .2
Benign Breast Disease	20	4. 2
Oral Contraceptives	0	0.0
Early menarche	8	1.7
Late menopause	7	1. 5
No childbirth	32	6. 8
No breastfeeding/breastfed	41	8. 6
Radiation exposure	24	5.1
Alcohol	31	6.3
Smoking	50	10. 5
Fatty diet	13	2. 7
Obesity	8	1. 7
Coins under breasts*	**95**	**20.0**
Tight/Dirty brassiere*	**3**	**0. 6**
When husband sucks the breasts*	**28**	**5. 9**

Respondents have poor knowledge of the Risk factors for breast cancer with high levels ofmyths

*Indicates incorrect responses – myths −31.9%)

This same trend was noted specifically about the knowledge of signs and symptoms of the disease which was generally found to be very low. As described in Table 3, specific signs and symptoms mentioned by the respondents include in changes in breast size and shape (15.6%), breast lump (46.6%) nipple discharge (13.1%) nipple retraction (5.5%), palpable axillary lymph nodes (0.4%). Generally, respondents with higher education performed better (U=3138, N=474, p

**Table 3:** Knowledge of breast cancer signs and symptoms

	Correct Responses		Incorrect Responses	
	
Sign and Symptom	Quantitative N=474	Qualitative N=20	Quantitative N=474	Qualitative N=20
Changes in the Size and Shape of the breast	74	2	400	18
Nipple Retraction	26	3	448	17
Nipple Discharges	62	1	412	19
Pains in the Breast	22	2	452	18
Lump/Mass in the Breast	221	5	253	15
Lump in the Armpit (Axilla)	2	0	472	20
Swelling of Breast	15	3	459	17
Sore of the Breasts	21	1	453	19

As shown in Table 3, Respondents’ knowledge of the signs and symptoms of breast cancer was generally found to be very low. Specific signs and symptoms mentioned by the number of the respondents are indicated.

### Breast Screening Practices

The self-reported breast cancer screening (breast self-examination 32%, clinical breast examination 12% and mammogram 2%) rates among the respondents were also found to be very low. As with the knowledge about the disease, higher educational of the respondents was very significant for breast cancer screening practices (SBE- X^2^ =43.797, df=4, p^2^ =25.786, df=4, ^2^ =9.954, df=4,

### Attitude towards breast cancer and screening

The respondents' attitudes towards the disease include fear which was linked to death in most cases; denial and guilt; as well as the spiritual and supernatural attributes of the disease. The respondents not only linked the disease with death but also linked surgical treatment of the disease with death. This is because about 60% of the patients present for treatment with advanced stages of the disease and as such many patients die shortly after surgical intervention. The women displayed a high level of reliance on God for protection from the disease, as well as on divine intervention and healing [19] and cited several reasons for non- participating in screening mammography as presented in Table 4.

**Table 4:** Reasons for non-participating in mammography screening

Reason Given n=474	Number	Percentage
Unaware of facilities for mammographic screening in the Community	159	33.5
Mammographic screening was not necessary because they did not have cancer	103	21.7
Because they were not referred by their doctors	82	17.3
Financial constraints	128	27.0
Absence of family history of the disease	24	5.1
Afraid to find out if they had cancer	19	4.0
Did not want to expose their bodies	5	1.1
Afraid of the procedure	5	1.10
Afraid of the effects of x-rays	5	1.1
Because of the negative attitude of health workers	1	0.1

In addition to the above, we reproduce some of responses gathered from the interviews with breast cancer patients, herbalists and the Consultants to highlight the socio-economic factors affecting breast cancer in Ghana.

*“I am paying for the treatment with money from my children and some donations from my Church...my children would not allow me to go for herbal treatment”*- Patient

“The cost of treatment and screening is very expensive and therefore a lot of women cannot afford; then it would be helpful if centres were opened where women can walk in for a mammogram for a small fee especially for the low-income earners”- Patient

*“The treatment has brought a very serious financial burden to my family and my husband had to take a loan to supplement family resources but that was not even enough to pay for the full cost of treatment”*-Patient

*“The Government should consider subsidizing the cost of treating the disease because a lot of women in Ghana die of the disease because they could not afford to pay for the high cost of treatment”*-Consultant

*“You see the breasts are very important to every woman without which, a woman is never complete and no man would be happy to marry a woman whose breast is removed and I would prefer to die than to live without my breasts”*-Patient

*“God's intervention is the only means that protection from the disease could be achieved. Whatever is said in the Bible would come to pass and the fact that scientists have not been able to come out with the actual causes of the disease points to the spiritual nature of the disease”*-Patient

*“Cancer is “Obosam disease” (devil's disease) and for which reason doctors are unable to treat the disease”*-Herbalist

*“Cancer is a supernatural disease and can only be treated by spiritual powers but not by doctors in the hospitals”*-Herbalist

*“Some believe it is an act of God, a family disease, an act of the devil or a curse; others people see the disease as someone's fault”*-Consultant

*“Another problem is the fear of removal of the breasts which has several social and marriage implications with the husbands and which prevents the women from reporting to the hospitals”*-Consultant

*“Many women cannot afford the cost of mammogram in Ghana which is about 400,000 in Private Health Institutions and around 250,000 in Public Health Institutions, considering the fact that the average basic salary is about 500,000 a month”*-Consultant

As could be seen, financial constraint was described as a major obstacle to accessing health care in general and breast cancer care in particular. This conclusion was drawn from the reported difficulty, encountered by many of the respondents, in paying for the cost of care and which was also corroborated by the Consultants. As noted that attitude towards breast cancer and breast screening practices among the respondents were varied including fear, denial and guilt.

## Discussion

This study provided new insights into the perceptions, knowledge, beliefs, attitudes, and practices of the Ghanaian women in Accra and Sunyani with respect to breast cancer and the breast cancer screening programmes.

### Respondents’ sources of health information

Respondents mentioned mass media as their main sources of health information. Health workers as well as families/friends were deemed ineffective or less effective as summarized in Table 1 The data strongly suggested the usefulness of exposure to multiple sources. However, this is in sharp contrast with a study in the United Kingdom where as many as 92% of the general population mentioned that General Practitioners were the main source of health information [20] In Ghana, doctor-patient ratio is far below the standard set by the World Health Organisation (WHO). Statistics indicate that the doctor-patient ratio in Ghana is 1:13,000, a figure far below the WHO global standard pegged at 1:5,000 making access to doctor very difficult [10]. On the other hand, access to the General practitioner (GP) in the UK is much easier. However, media especially the local FM radio stations are very accessible in Ghana. These radio stations carry health care advice in Ghana whereas this is not a big feature in the UK. Despite the overwhelming evidence of mass media effectiveness in raising awareness, increasing knowledge and changing attitudes and behaviour, it could create a big problem for the society if inaccurate information is disseminated through the media. It is therefore important that any information put on the media should be accurate so that the target population would not be given inaccurate information about the disease.

### Knowledge of the respondents on breast cancer

UN Human Development Index Report [21] puts the National Female Literacy rate in Ghana at 51%. The level of education of the respondents in the present study (57%) was well above this average. However, the respondents were poorly informed about the risk factors and the signs and symptoms of the disease. This was evident from the poor scores from the questions on the signs and symptoms as well as the risk factors of the disease; coupled the high levels of myths as shown in Table 2 and Table 3. When properly employed, the mass media which was identified as the main source of health information, could be used create the needed awareness and education about the disease in Ghana. Overall, respondents with higher education performed better, with more correct responses.

### Respondents’ perception of the incidence and prevalence of breast cancer in Ghana

Nearly all the participants in the study acknowledged that the number of breast cancer cases was increasing. The absence of cancer registries in Ghana makes it extremely difficult to determine the actual incidence and prevalence of the disease. This is due to the fact no previous studies have been done in Ghana and also the fact the available figures are from individual hospitals which are under reported. In order to make a strong case for more resources to be spent on cancer in Ghana, and ensure cost-effectiveness, it is important that the actual disease burden is known. In addition to the increasing numbers of patients, it is also worrying that Ghanaian women tend to get breast cancer at a younger age [8,9,22] a view also shared by the 3 Consultants who participated in the study.

### Attitudes of the respondents towards breast cancer

The attitudes towards the disease ranged from fear which was linked to death in most cases; denial and guilt; as well as the spiritual and supernatural attributes of the disease. The respondents not only linked the disease with death but also linked surgical treatment of the disease with death. This is because many women present for treatment with advanced stages of the disease and as such many patients die shortly after surgical intervention.

The women displayed a high level of reliance on God for protection from the disease, as well as on divine intervention and healing. For example, a respondent in a response to a question on the causes of the disease said *“since the cause of the disease appears unknown one is tempted to believe that is due to some spiritual cause and curses”*. Another respondent remarked that “the disease is an act of God”. *“Nothing happens without a cause and since nobody knows the cause of the disease it would not be out of place to attribute the disease to these supernatural forces”* was another respondent. *“My main problem is how my husband would continue to love and accept me after my breast has been removed”* an obviously this is a case of denial and guilt. In the interviews with the Consultants, it came quite strongly that breast cancer patients delay in reporting to the hospital because they were afraid that their husbands will no longer accept them after mastectomy.

### Social-cultural influences on breast cancer

In Ghana, as in most developing countries, women appear to take a lower priority in society than men, leading to the management of female related cancers, such as breast cancer, being underfunded and lacking the urgency for the screening and treatment seen in many western countries [23,24]. It is therefore important to involve husbands in cancer educational programmes so that they support their wives and prevail on them to adopt early detection activities, in order that they seek appropriate early treatment [25].

### Healthcare services system

The inequitable geographical distribution of health facilities and services in Ghana in favour of the cities has long been recognized as a major problem to the health delivery system [26]. However, in the current study, there were no significant differences both in the attitude towards breast cancer and breast screening practices among the respondents from Accra, the National Capital and Sunyani, the semi- urban Capital of Brong Ahafo Region.

### Cost of breast cancer care in Ghana

The discussion of breast cancer care in Ghana will be incomplete without considering the economic barriers that prevent Ghanaian women from seeking appropriate screening and treatment. This is because costs for diagnosis and treatment was strongly expressed by many women who participated in the study as a major barrier to breast cancer care in Ghana, a concern also corroborated by the medical consultants who participated. Breast cancer patients in Ghana are required to make out-of-pocket payments to pay significant part of the cost of screening, diagnosis and treatment at the point of service. However, this system has great potential to negatively affect access to health care, especially for women.

### Traditional medicine and breast cancer care

A common observation from the traditional healers interviewed in the study was their claim to treat every condition including breast cancer and they were not particularly modest in their self-claimed abilities. Another disturbing phenomenon was the unwillingness on the part of the traditional healers to refer breast cancer patients to the hospitals based on their sense of supremacy over conventional medical practitioners in dealing with the disease.

## Conclusion

The study has revealed not only the poor knowledge of breast cancer and the screening methods but also the low of level breast cancer screening practices among the women who participated. This poor knowledge of the disease would lead to a low priority in early detection measures with its related poor outcome of the disease. It is therefore important to put in measures to provide breast cancer information in comprehensible manner to the benefit of the entire Ghanaian women population especially those in the low socioeconomic status and the rural communities.

Although some awareness activities are taking place in Ghana, it is important to question the tools which are being utilized to educate Ghanaian women about breast cancer and how effective these activities have been. For this reason, the study proposes the theory of planned behaviour as a model for planning and implementing breast cancer education and awareness programmes to improve the outcome of the disease in Ghana [27–30].

The study also came to the realization that the sophisticated strategies for breast cancer care in the developed countries such as routine mammographic screening cannot be implemented in Ghana [31]. This is because the country lacks the capacity in both human and material resources to effectively implement such programmes. In line with the purpose of the study, that is, develop an appropriate socio-economic and cultural specific model to improve breast cancer in Ghana, the study proposes a model as a framework for breast care on Ghana in particular and the developing world in general. The first approach to the model is to increase awareness and encourage the women to undertake BSE and report any suspicious findings for clinical evaluation. The second is to encourage wide spread adoption of CBE. The few mammogram centres can then be used for diagnostic purposes and screening for high risk or symptomatic women. Provision of treatment facilities and development of an efficient early referral system are stressed in the proposed model.
